# The multifaceted roles of mitochondria in osteoblasts: from energy production to mitochondrial-derived vesicle secretion

**DOI:** 10.1093/jbmr/zjae088

**Published:** 2024-07-01

**Authors:** Joonho Suh, Yun-Sil Lee

**Affiliations:** Department of Molecular Genetics, School of Dentistry and Dental Research Institute, Seoul National University, Seoul 08826, Republic of Korea; Department of Molecular Genetics, School of Dentistry and Dental Research Institute, Seoul National University, Seoul 08826, Republic of Korea

**Keywords:** Mitochondria, Osteoblasts, Bone formation, ATP, Mitochondrial-derived vesicles (MDVs), Extracellular mitochondrial secretion, Oxidative phosphorylation, Glycolysis, Oxidative stress, Mitophagy

## Abstract

Mitochondria in osteoblasts have been demonstrated to play multiple crucial functions in bone formation from intracellular adenosine triphosphate production to extracellular secretion of mitochondrial components. The present review explores the current knowledge about mitochondrial biology in osteoblasts, including mitochondrial biogenesis, bioenergetics, oxidative stress generation, and dynamic changes in morphology. Special attention is given to recent findings, including mitochondrial donut formation in osteoblasts, which actively generates mitochondrial-derived vesicles (MDVs), followed by extracellular secretion of small mitochondria and MDVs. We also discuss the therapeutic effects of targeting osteoblast mitochondria, highlighting their potential applications in improving bone health.

## Introduction

Mitochondria, traditionally known for their role in energy production, play diverse and crucial functions in cellular maintenance. One of the major characteristics of mitochondria is their ability to continuously alter their morphology through fusion and fission, which significantly impacts cellular behaviors. The dynamic nature of mitochondria is also driven by mitophagy, a selective degradation of damaged mitochondria central to mitochondria quality control. Recent discoveries have revealed that mitochondria can be extracellularly secreted by various cells, including astrocytes, bone marrow mesenchymal stem cells (BMSCs), and monocytes, adding complexity and diversity to mitochondrial biology.^1^

Bone formation is a fundamental process in vertebrate skeletal development, in which osteoblasts synthesize and deposit organic matrix (collagen) that is subsequently mineralized to form bones. It is an energy-intensive process that requires the significant involvement of mitochondria in osteoblasts. In addition to generating energy, mitochondria were reported to directly regulate osteogenesis through their extracellular secretion, which promoted the differentiation of osteoprogenitors,^2^ and through mitophagy, which mediated mineral deposition in bone collagen.^3^ Furthermore, our group recently demonstrated that mitochondrial donut formation, a distinct morphological feature observed during physiological osteogenesis, triggered mitochondrial-derived vesicles (MDVs) generation, and that modulation of mitochondrial morphology toward fission and donut formation enhanced osteoblast maturation.^2^ The strong link between mitochondria and osteogenesis has spurred the development and assessment of various mitochondrial-targeting agents to enhance osteoblast function. Here, we review the multifaceted roles of osteoblast mitochondria and their impact on osteoblast function and bone formation. We cover topics including mitochondrial biogenesis, bioenergetics, oxidative stress production, and dynamic changes in morphology during osteoblast differentiation. Finally, we also review current therapeutic strategies targeting osteoblast mitochondria and discuss their potential applications in promoting osteogenesis.

## Bioenergetics and mitochondrial biogenesis: OXPHOS vs glycolysis

The initial metabolic study performed on bone tissues of mice in the 1960s suggested that bones predominantly utilized glycolysis rather than oxidative phosphorylation (OXPHOS) for their metabolism.^4^ The study reported that compared to other tissues such as the liver, bones exhibited lower oxygen consumption and significantly higher glucose consumption, leading to the abundant production of lactate. Similar findings were observed in rat calvaria,^5^ emphasizing the importance of glycolysis in bone metabolism. The significance of glycolysis in the regulation of bone mass was further supported by recent data suggesting defective glucose metabolism as the major cause of diabetes-induced bone loss, which showed that elevating glycolysis through administration of metformin or osteoblast-specific overexpression of *Hif1a* (hypoxia inducible factor 1 subunit alpha) or *Pfkfb3* (6-phosphofructo-2-kinase/fructose-2,6-biphosphatase 3) in mice improved bone mass in diabetic mice.^6^^,^^7^ In vivo tracing with a stable isotope also demonstrated that glucose was largely metabolized to lactate and minimally contributed to the tricarboxylic acid (TCA) cycle in mouse cortical bones.^8^

Although glycolysis is evidently critical in the maintenance and growth of bone, evaluation of metabolic activity in isolated osteoblasts has revealed more complex and time-dependent results. Specifically, Komarova et al. closely examined changes in metabolism in differentiating primary calvarial osteoblasts in vitro and showed that OXPHOS was the primary energetic pathway utilized during the early stages (before day 10) of differentiation, but a shift to glycolysis occurred during the later stages (after day 10) when mineralized nodules formed.^9^ Similarly, Guntur et al. utilized a Seahorse real-time metabolic analyzer to measure oxygen consumption rate (OCR), indicative of OXPHOS, and extracellular acidification rates, indicative of glycolysis in mouse calvarial osteoblast cell line MC3T3-E1 and demonstrated that both OXPHPOS and glycolysis were initially upregulated in differentiating osteoblasts, but glycolysis was favored during the later stages of differentiation.^10^^,^^11^ Using the same method, Lee et al. showed that the contribution of glycolysis in total adenosine triphosphate (ATP) generation increased from approximately 40% at day 0 to 80% at day 7 of differentiation in primary calvarial osteoblasts.^8^ Our group also recently reported that both OCR and glycolytic proton efflux rate were elevated during the early maturation steps, but only glycolysis remained upregulated, while mitochondrial respiration decreased during the later mineralizing periods in primary calvarial osteoblasts.^2^ Noteworthily, mitochondrial ATP production may be critical during the early osteoblast maturation period since treatment with oligomycin A (ATP synthase inhibitor), antimycin A (mitochondrial complex III inhibitor), or siRNA targeting *Atp5a1* gene, which encodes the alpha subunit of the mitochondrial ATP synthase F1 complex, at the initiation of osteogenic induction significantly impaired osteoblast differentiation.^2^ A recent review of metabolic activities in osteoblasts has underscored that OXPHOS increases as osteoblast maturation advances, although there are ongoing debates regarding the extent of glycolysis levels during osteoblast differentiation.^12^ Although further investigation is needed, particularly considering the distinct origins of osteoblasts (calvaria vs long bones), current evidence suggests that differentiating osteoblasts actively utilize both OXPHOS and glycolysis during the initial phases but tend to favor glycolysis as maturation progresses.

Because OXPHOS far more efficiently produces ATP than aerobic glycolysis, why mature osteoblasts preferentially utilize glycolysis has remained unclear. It has been suggested that aerobic glycolysis is upregulated to offset the increase in ROS generation by OXPHOS.^13^ Additionally, Guntur et al. demonstrated that the increase in glycolytic ATP production in mature osteoblasts was the result of a decrease in mitochondrial membrane potential and OXPHOS activity.^10^ Similarly, using calvarial osteoblasts in which mitochondria were endogenously labelled with GFP, our group showed that mitophagy dramatically increased after differentiation day 14. This was confirmed by the increase in GFP-positive mitochondria co-localized with autophagic-lysosomal markers, LAMP1 (lysosomal-associated membrane protein 1) or LC3 (microtubule-associated protein light chain 3), suggesting that the decreased number of mitochondria after massive mitophagy may be the direct cause of reduced OXPHOS during the late differentiation stages.^2^

Consistent with the early increase in OXPHOS, mitochondrial biogenesis was found to increase during the initial phases of osteoblast maturation. Protein expression levels of mitochondrial biogenesis markers peroxisome proliferator-activated γ receptor coactivator-1α (PGC-1α) and mitochondrial transcription factor A (mtTFA or TFAM) were upregulated and peaked at day 7 of differentiation in MC3T3-E1 osteoblasts.^14^ Our group also reported that the expression of *Ppargc1a*, encoding PGC-1α, peaked at day 3, while the expressions of *Tfam*, *Cox4i1* (cytochrome c oxidase subunit 4I1), and *mt-Cytb* (cytochrome B) peaked at day 7 in primary calvarial osteoblasts.^2^ Mitochondrial DNA copy number and expressions of mitochondrial proteins, COX4, and translocase of outer mitochondrial membrane 20, also peaked at day 7, after which the levels gradually declined possibly due to the progressive increase in mitophagy.^2^ Furthermore, enhancing mitochondrial biogenesis appears to accelerate osteogenic differentiation. For instance, An et al. demonstrated that activation of Wnt signaling pathway in C3H10T1/2 mouse mesenchymal cells promoted their osteoblastic differentiation through stimulating mitochondrial biogenesis.^15^ Increasing mitochondrial biogenesis through overexpression of *Tfam* enhanced Wnt-induced osteogenesis, while suppressing mitochondrial biogenesis through treatment with Zidovudine significantly impaired the osteogenic activity of C3H10T1/2 cells.^15^ In addition, nicotinamide (NAM), a form of vitamin B3, was recently shown to promote the differentiation of MC3T3-E1 osteoblasts partly through increasing the expression of *Ppargc1a* and mitochondrial biogenesis.^16^ These results suggest that potential bone therapeutics may target mitochondrial biogenesis in osteoblasts.

Controversies persist regarding the metabolic activities during osteogenic differentiation of BMSCs. Shum et al. reported that OXPHOS was significantly enhanced during osteogenic differentiation of human MSCs, while glycolysis was unchanged and more critical during the proliferation of undifferentiated MSCs.^17^ They described that calvarial osteoblasts and MSCs exhibit distinct metabolic characteristics due to their different embryonic origin.^17^ Contrastingly, Ma et al. demonstrated that activating glycolysis through treatment with rotenone (complex I inhibitor) significantly promoted the osteogenic differentiation of BMSCs in mice, while inhibiting glycolysis through treatment with dichloroacetate, sodium oxamate, or 2-deoxy-D-glucose significantly impaired their osteogenic differentiation, highlighting the significance of glycolysis in MSC-mediated osteogenesis.^18^ They also showed that extracellular vesicles delivered lactate dehydrogenase A into BMSCs and stimulated glycolysis, enhancing bone formation in vivo.^18^ Further investigations are needed to clarify whether metabolic characteristics in osteogenic cells are embryonic origin-specific (neural crest vs mesoderm-derived) and differ between intramembranous and endochondral bone formation. A deeper understanding of osteogenic metabolism will not only advance insights into the mechanisms of metabolic bone diseases but also accelerate the development of strategies that modulate mitochondrial metabolism to promote osteogenesis.

## Oxidative stress

Mitochondria produce most of the cellular ROS through OXPHOS, during which oxygen is reduced to superoxide anions that are subsequently converted to additional ROS such as hydrogen peroxide (H_2_O_2_). Accumulation of ROS is normally counterbalanced by the endogenous antioxidant defense system involving both non-enzymatic antioxidants, including ascorbic acid, vitamin E, and glutathione, and enzymatic antioxidants, including superoxide dismutases (SODs), catalase, glutathione peroxidases, and peroxiredoxins. Endogenous ROS, when the levels are controlled, may behave as second messengers to activate signaling pathways related to proliferation, differentiation, and survival, but excessively high levels of ROS can cause multiple pathological conditions, including osteoporosis.^19^

Conflicting views exist regarding the impact of endogenous ROS on osteoblast differentiation, although many studies agree with the negative effect of dysregulated ROS on bone. During osteogenic differentiation of MC3T3-E1 osteoblasts, Arakaki et al. reported that endogenous ROS levels increased in a time-dependent manner, and suppressing it through treatment with the antioxidant N-acetylcysteine (NAC) dose-dependently impaired osteoblast mineralization.^20^ In contrast, Gao et al. indicated that endogenous ROS and mitochondrial protein oxidation levels were largely decreased during MC3T3-E1 osteoblast differentiation.^14^ Yamada et al. also demonstrated that NAC enhanced bone regeneration and osteogenic differentiation of rat BMSCs.^21^ Our group also assessed the endogenous levels of mitochondrial ROS in primary mouse osteoblasts that showed a steady increase after the induction of differentiation.^2^ However, we observed that ascorbic acid, also known as vitamin C, an essential cofactor for proline and lysine hydroxylases required to stimulate in vitro osteogenesis, facilitated osteogenic differentiation but also decreased mitochondrial ROS levels upon its treatment.^2^ Further investigation is needed to confirm whether the antioxidant effect of vitamin C directly contributes to osteoblast differentiation. Gao et al. showed that the expression and activity of SOD2, an antioxidant enzyme that specifically localizes in mitochondria, increased in mouse osteoblasts during differentiation to positively regulate osteogenesis by controlling oxidative stress.^14^ The group showed that downregulation of either *Sod2* or mitochondrial deacetylase sirtuin 3 (*Sirt3*) elevated mitochondrial ROS and significantly blunted osteoblast differentiation. A similar outcome was recently reported, suggesting that NAM stimulated MC3T3-E1 osteoblast differentiation through activation of SIRT3-FOXO3A axis and the resultant increase in the expression of antioxidant enzymes such as *Sod1* and *Sod2*.^16^ The group proposed that NAM could be a potential therapeutic treatment for ROS-induced bone disorders.

Further genetic studies in mice indicated that ROS overload could negatively impact bone mass. For instance, Schoppa et al. utilized Runx2-Cre to conditionally delete *Sod2* expression in osteoblast lineage cells in mice and demonstrated that *Runx2-Cre*; *Sod2^flox/flox^* mice displayed a significant decrease in bone mass compared to *Sod2^flox/flox^* mice at both 12 and 52 wk of age.^22^ Mitochondrial ROS accumulation through *Sod2* deletion in RUNX2-positive osteoblast precursor cells reduced their osteogenic potential and stimulated their adipogenic differentiation.^22^ Moreover, *Sod2* deficiency elevated the expressions of senescence-related markers, *p21* and *p16^INK4a^*, and senescence-associated secretory phenotype factors, interleukin 6 and tumor necrosis factor α, in osteoblasts.^22^ These findings suggest that dysregulation of mitochondrial oxidative stress can have detrimental effects on osteoblast function, and enhancing antioxidant defense system may be a promising therapeutic approach for ROS-related bone loss.

## Mitochondrial dynamics: morphological changes, extracellular secretion, and mitophagy

In addition to generating ATP, mitochondria exhibit unique features in which they continuously undergo fusion and fission, distinct morphological changes, MDVs generation, extracellular secretion, and removal through mitophagy.^1^^,^^23^ These processes are collectively known as mitochondrial dynamics and are critical for proper cellular function and differentiation.

### Fusion and fission

One of the initial studies that closely examined mitochondrial morphology in MC3T3-E1 calvarial osteoblasts reported that mitochondria existed as a continuous reticulum (long tubular shape) in the non-differentiated state (day 0) but were fragmented after osteogenic differentiation.^20^ Specifically, the study demonstrated that fragmented mitochondria increased from approximately 8% (day 0) to 18%, 23%, and 40% at days 5, 10, and 14, respectively, while long tubular mitochondria decreased from approximately 18% (day 0) to 3%, 4%, and 2% at days 5, 10, and 14, respectively.^20^ Moreover, reducing mitochondrial fragmentation and simultaneously increasing mitochondrial elongation significantly impaired the mineralizing activity of osteoblasts.^20^

Controversy exists regarding the effects of modulating mitochondrial morphology in osteogenesis, which may be context-dependent. For instance, as opposed to Arakaki et al. that showed the positive effect of mitochondrial fragmentation on osteoblast differentiation, Gan et al. illustrated that inhibiting mitochondrial fragmentation through blocking dynamin-related protein 1 (DRP1), a key promoter of fission, improved the osteogenic differentiation of human osteosarcoma cell line exposed to oxidative stress.^24^ Also, diminishing mitochondrial fission through treatment with mitochondrial division inhibitor Mdivi-1 significantly rescued an inflammation-induced decrease in MC3T3-E1 osteoblast differentiation.^25^ In contrast, a recent investigation by Menale et al. demonstrated that addition of palmitic acid (PA) enhanced mitochondrial fragmentation and osteogenic differentiation of MC3T3-E1 cells and primary mouse osteoblasts, while preventing mitochondrial fission through Mdivi-1 treatment impaired osteogenesis.^26^ In agreement with Arakaki et al., our group recently showed that mitochondrial fragmentation significantly increased during mouse osteoblast maturation and interrupting this process through downregulation of mitochondrial fission 1 (*Fis1*) or treatment with mitochondrial fusion promoting chemical M1 impaired osteogenesis.^2^ To overcome the limitation of the popular enzymatic method of primary osteoblast isolation which yields a heterogenous cell population containing fibroblasts and lymphocytes, our group purified osteoblasts through FACS in genetically engineered mice in which GFP is expressed specifically in the mitochondrial matrix of osteoblasts. Using the pure osteoblast population, our group demonstrated that promoting mitochondrial fragmentation through genetic knockdown of optic atrophy 1 (*Opa1*) or overexpression of *Fis1* significantly accelerated osteogenic activity in osteoblasts, suggesting that mitochondrial fission is a critical feature of osteoblast maturation under physiological conditions.^2^ Notably, the studies that showed positive osteogenic effects upon inhibiting mitochondrial fission were evaluated on dysfunctional osteoblasts exposed to oxidative stress or inflammation.^24^^,^^25^ Whether mitochondrial morphological characteristics of osteoblasts are distinct between physiological and pathological conditions requires further examination.

### Mitochondrial donut formation and MDVs generation

A noteworthy morphological feature of mitochondria observed during osteoblast maturation is the formation of a donut-like structure. This distinctive morphology, characterized by a ring-shaped or toroidal appearance, has been documented in osteosarcoma,^27-29^ osteogenically-stimulated MC3T3-E1 cells,^20^ and more recently, in mature primary osteoblasts by our group (Table 1).^2^ Interestingly, rather than forming through the bending of the extended mitochondrial membranes around the cytoplasm, as previously suggested in mouse embryonic fibroblasts (MEFs) and Chinese hamster ovary cells,^30-33^ many donut-shaped mitochondria in osteoblasts appeared to be generated through the formation of a hole near the center of a circular/globular mitochondrion, as shown through live imaging of osteoblasts using the lattice structured illumination microscopy.^2^ Donut-shaped mitochondria in osteoblasts also appeared smaller (mostly less than 1 μm in diameter) than those previously reported in other cells (with an average diameter of ~1.3 μm).^34^

**Table 1:** Mitochondrial donut formation and suggested mechanisms.

Cell/Tissue	Experimental set-up	Morphological nomenclature	Mechanisms of donut formation and associated conditions	References
Avian retinal pigment epithelial cells	Normal Continuous light exposure	Ring/Donut	Possibly formed to increase the surface area for interaction with the cytoplasm	Lauber^52^
MRC5 human lung fibroblasts	Rotenone (complex I inhibitor) treatment	Donut	Formed by a decrease in mitochondrial membrane potential Formed when cellular respiration rate is decreased by about 40%	Benard et al.^53^
143B human osteosarcoma cells	mtDNA depletion by EtBr	Ring	Formed by metabolic changes due to lack of mtDNA-coded proteins May represent an adaptive change to altered oxygen levels	Ferraresi et al.^28^
Muscle cells of *C. elegans*	Overexpression of a dominant-negative form of DRP-1	Interconnected and dilated	Formed by inhibition of mitochondrial fission	Tan et al.^54^
N27 rat neuronal cells	Overexpression of PINK1 or MFN2	Donut	Formed in response to enhanced mitochondrial fusion or decreased fission	Cui et al.^55^
H9c2 rat cardiomyoblasts, primary rat cardiomyocytes, RBL-2H3 rat basophilic leukemia cells, MEFs, and HeLa cells	Hypoxia or FCCP (OXPHOS uncoupler) treatment	Donut	Formed by opening of the mPTP or K^+^ channels and mitochondrial depolarization Not induced by ATP depletion (by oligomycin) or overexpressing OPA1 or a dominant-negative form of DRP1	Liu and Hajnoczky^34^
CHO cells	Inhibitors of caspases 8 (z-IETD) or 9 (z-LEHD)	Loop/Donut	Formed by intra-mitochondrial fusion	Peng et al.^31^
MEFs	CCCP treatment	Spheroid with a lumen (hollow sphere), ring- or C-shaped	Formed by oxidative damage and not by mitophagy	Ding et al.^33^
MEFs, HEK-293 cells, HeLa cells, and mouse liver	CCCP or sodium azide (complex IV inhibitor) treatment Acetaminophen overdose in mice	Spheroid, ring-shaped	Formed in response to ROS MFN1 and MFN2 required Formation inhibited by Parkin Not related to MDVs	Ding et al.^37^
Mouse skeletal muscle	Normal	Donut	Found more commonly in glycolytic muscle than oxidative or cardiac muscle	Bleck et al.^56^
SK-KOSA, MC-KOSA, and BW-KOSA canine osteosarcoma cells	Normal	Donut	Formed by reductions in mtDNA, electron transport chain proteins, and mitochondrial respiration	Jackson et al.^29^
Primary mouse cardiac progenitor cells	BNIP3L and FUNDC1 knockdown	Donut	Formed by disruption of mitophagy	Lampert et al.^57^
U2OS human osteosarcoma cells	Serum starvation FCCP treatment	Donut	Formed by opening of mPTP or K^+^ channels Resist mitophagy Sufficient mitochondrial length required (most greater than 1.5 μm in length prior to donut formation)	Zhou et al.^58^
Human MECs	Cells cultured on stiff fibronectin-coated ECM (60 k Pa)	Toroidal	Formed by stiff ECM, β1 integrin gain-of-function, hyperglycemia, mitochondrial Ca^2+^ overload, or mitochondrial ROS Show elevated mitochondrial membrane potential	Tharp et al.^35^
Primary mouse calvarial osteoblasts and mouse calvaria	Normal bone tissue Osteogenic differentiation in vitro	Donut	Actively generate MDVs Formation increased by *Opa1* knockdown or *Fis1* overexpression Possibly formed by a mild reduction in mitochondrial respiration	Suh et al.^2^

Abbreviations: CCCP, carbonyl cyanide 3-chlorophenylhydrazone, OXPHOS uncoupler; CHO, Chinese hamster ovary; ECM, extracellular matrix; EtBr, ethidium bromide; FCCP, carbonyl cyanide 4-trifluoromethoxyphenylhydrazone, OXPHOS uncoupler; HEK, human embryonic kidney; MDVs, mitochondrial-derived vesicles; MECs, mammary epithelial cells; MEFs, mouse embryonic fibroblasts; mPTP, mitochondrial permeability transition pore; mtDNA, mitochondrial DNA; NAC, N-acetylcysteine; OXPHOS, oxidative phosphorylation.

The precise mechanism of mitochondrial donut formation and its functional role in osteoblasts remain uncertain. Although a decrease in mitochondrial membrane potential was suggested to be associated with mitochondrial donut formation in MEFs,^32^ mitochondrial membrane potential was not largely reduced in donut-shaped mitochondria in mouse osteoblasts.^2^ Mitochondrial ATP production levels were decreased in osteosarcoma cell lines displaying abundant donut-shaped mitochondria, implying that a reduction in energy production may induce mitochondrial donut formation.^27-29^ In support of this theory, reducing ATP production in mouse osteoblasts through treatment with bongkrekic acid, a mitochondrial adenine nucleotide translocator inhibitor, significantly enhanced mitochondrial donut formation.^2^ Furthermore, suppressing mitochondrial ATP production through treatment with oligomycin A, antimycin A, or si*Atp5a1* significantly increased mitochondrial donut formation in osteoblasts, but donut-shaped mitochondria formed this way were largely swollen and did not promote osteoblast maturation, unlike those generated during the normal osteoblast differentiation process, suggesting that other mechanisms for physiological mitochondrial donut formation in osteoblasts likely exist.^2^ It is possible that mitochondrial donut formation in osteoblasts is influenced by the extracellular matrix (ECM) through integrin-mediated mechanosignaling. This mechanism was previously suggested in human mammary epithelial cells located within stiffened tumor microenvironments, which displayed fragmented/toroidal mitochondria similar to those in osteoblasts.^35^ Furthermore, mitochondrial donut formation in osteoblasts appears to be regulated in part by mitochondrial fusion and fission genes including *Opa1* and *Fis1* since downregulation of *Opa1* or overexpression of *Fis1* significantly elevated mitochondrial donut formation along with mitochondrial fission.^2^ However, downregulation of *Dnm1l*, encoding DRP1, also increased mitochondrial donut formation despite promoting mitochondrial elongation, suggesting that mitochondrial donut formation and fragmentation are not always parallel.^2^ Donut-shaped mitochondria may also form in response to increased mitochondrial ROS, as blocking ROS prevented donut formation in various cells.^35-37^

Notably, donut-shaped mitochondria in osteoblasts actively generated MDVs,^2^ which are nanoscale vesicles (70–100 nm in diameter), smaller than microvesicles (100–1000 nm in diameter), and are surrounded by single or double membranes derived from the outer and/or inner mitochondrial membrane. Unlike exosomes (30–150 nm in diameter) known to originate from multivesicular bodies, MDVs bud directly from the mitochondrial surface and are generally produced in a variety of cells for intercellular communication or clearance of damaged mitochondrial parts by fusing with lysosomes.^1^ Currently, definitive membrane markers specific to MDVs, exosomes, and other extracellular vesicle subtypes are not clearly established and require further investigation. Despite their prevalence in various cells, the role of MDVs in osteogenic cells remains largely unknown. However, a recent study demonstrated that the altered composition of MDVs, which are subsequently degraded by lysosomes, contributes to aging-related decline in osteogenic capacity of mouse MSCs, implying active involvement of MDVs during osteogenesis.^38^ Moreover, in osteoblasts, MDVs released by donut-shaped mitochondria were secreted into the extracellular space, promoting the differentiation of osteoprogenitors by delivering cargo proteins.^2^ Approximately half of the extracellular MDVs secreted by mature osteoblasts were composed of only the outer mitochondrial membrane.^2^ However, the difference in the mechanisms and functions between MDVs with a double mitochondrial membrane and only the outer mitochondrial membrane is yet to be determined. Additionally, whether the formation of donut-shaped mitochondria provides a geometrical or energetic advantage for MDV production over linear mitochondria requires further investigation. Exploring these aspects in future studies may uncover unique genetic regulation mechanisms and functional aspects of donut-shaped mitochondria and MDVs in osteoblasts.

### Extracellular mitochondrial secretion

**Figure 1:**
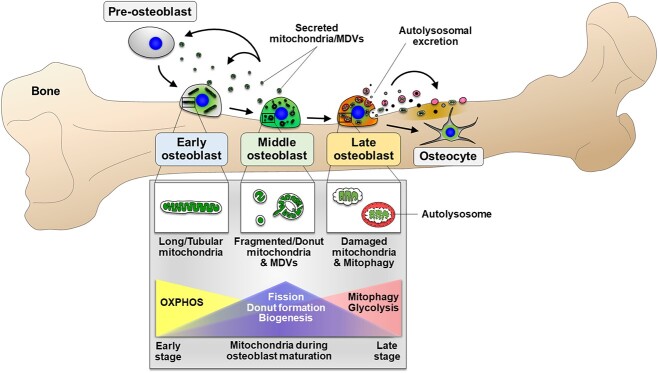
Overview of the functions and mitochondrial characteristics of maturing osteoblasts. Early osteoblasts are characterized by abundant long and tubular mitochondria, while more mature (middle) osteoblasts exhibit fragmented or donut-shaped mitochondria and MDVs. In the middle stage, osteoblasts actively secrete mitochondria and MDVs into the extracellular space, promoting the differentiation of pre-osteoblasts and early osteoblasts. Late-stage osteoblasts, on the other hand, engage in active mitophagy. This process mediates the release of vesicles or autolysosomal contents such as damaged mitochondrial components and mineral granules into the bone matrix. During the early stage, OXPHOS is elevated, while glycolysis is upregulated in the late stage of osteoblast maturation.

In 2023, our group reported for the first time that mitochondria in mature mouse osteoblasts were secreted into extracellular space (Figure 1).^2^ This exploration into the extracellular secretion of mitochondria in osteoblasts was motivated by the accidental observation of strong GFP signals in the bone matrix of genetically engineered mice that express GFP exclusively in the mitochondrial matrix and by earlier reports demonstrating the presence of abundant mitochondrial proteins in the bone matrix.^39^ We showed that during osteogenesis, mitochondrial fragmentation, donut formation, and cluster of differentiation 38 (CD38)/cyclic ADP-ribose (cADPR) signaling triggered the extracellular secretion of mitochondria that subsequently promoted the differentiation of osteoprogenitors through the transport of protein cargos.^2^ Treatment with osteoblast-secreted mitochondria significantly enhanced bone regeneration in mice, suggesting the potential of mitochondrial transplantation therapy for bone healing.^2^ Mitochondrial fission or donut formation appeared to be critical for extracellular mitochondrial secretion since reducing mitochondrial fusion through *Opa1* knockdown or promoting mitochondrial fission/donut formation through *Fis1* upregulation significantly elevated the extrusion of mitochondria into the extracellular space.^2^ CD38 is highly expressed in MC3T3-E1 osteoblasts^40^ and previously described to play significant functions during bone formation through an unknown mechanism.^41^ Our group partly uncovered this mechanism, demonstrating that CD38/cADPR signaling directly stimulated the extracellular secretion of mitochondria in osteoblasts. However, additional molecular pathways that control mitochondrial extrusion may exist and be identified in future studies. Additionally, further examination is required to determine the specific osteo-stimulating protein cargo carried by the secreted mitochondria and whether mitochondrial secretion has an extracellular effect on mineralization.

### Mitophagy

More than 50 yr ago, mitochondria in rat osteoblasts were shown to contain electron-dense calcium phosphate granules that may participate in bone formation.^42^ In 2012, Boonrungsiman et al. described that these calcium phosphate granules were transferred from the mitochondria to unidentified intracellular vesicles that mediate their release to the ECM for deposition on collagen fibrils.^43^ Several years later, Pei et al. revealed that mitophagy was triggered upon accumulation of amorphous calcium phosphates in the mitochondrial matrix and mediated the transfer of calcium phosphate granules to autolysosomes, after which the mineral granules were extracellularly released to initiate calcification.^3^ Pei et al. highlighted the importance of mitophagy in ossification by demonstrating that treatment with the mitophagy inducer carbonyl cyanide 3-chlorophenylhydrazone significantly elevated the mineral deposition of human dental pulp stem cells (hDPSCs), while exposure to the mitophagy inhibitors bafilomycin A1 or cyclosporin A impaired it.^3^ Downregulation of phosphatase-and-tensin homolog-induced putative kinase 1 (*Pink1*) also blunted osteogenic activity in hDPSCs.^3^ Our group recently analyzed mitophagy in mouse osteoblasts during osteogenic maturation and observed that protein expressions of mitophagy markers PINK1, Parkin, LC3, and LAMP1 were elevated at late stages (days 14 and 21) of maturation when mineralization activity became prominent,^2^ supporting the significant involvement of mitophagy in mineral deposition described by Pei and colleagues.^3^ However, we noticed that mitophagy also importantly regulated the early maturation of osteoblasts, which was shown through the impaired early alkaline phosphatase activity (at day 5) in response to siRNA-mediated downregulation of mitophagy-related genes or treatment with autophagy/mitophagy inhibitors, BafA1 and Lys05.^2^ The precise mechanism by which mitophagy regulates early osteoblast maturation steps before the onset of mineralization remains to be further examined.

## Therapeutic targeting of osteoblast mitochondria

**Figure 2:**
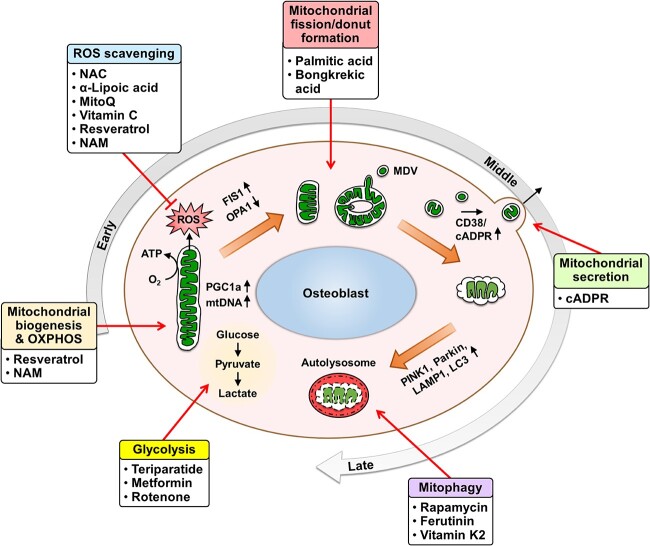
Mitochondrial modulators suggested to stimulate osteoblast differentiation. Molecules targeting mitochondrial biogenesis, OXPHOS, ROS, mitochondrial fission and donut formation, mitochondrial secretion, mitophagy, and glycolysis in osteoblasts are listed. Additionally, representative mitochondrial characteristics in early-, middle-, and late-stage osteoblasts are presented.

During osteogenesis, mitochondria in osteoblasts exhibit distinct signatures that may serve as therapeutic targets. Several key modulators of mitochondria were shown to stimulate osteoblast maturation by targeting mitochondrial metabolism, dynamics, or oxidative stress (Figure 2). Teriparatide, a widely prescribed bone anabolic protein consisting of amino acids 1 to 34 of human PTH, was shown to enhance bone formation by activating aerobic glycolysis and preventing glucose entry into the TCA cycle via IGF signaling in mouse osteoblasts.^44^ Suppressing glycolysis through treatment with dichloroacetate abrogated the bone mass increase induced by PTH.^44^ Metformin, the most prescribed antidiabetic agent, was documented to exert anabolic effects on bone partly through modulating osteoblast bioenergetics. In BMSCs isolated from type 2 diabetic mice, metformin treatment significantly promoted osteogenic differentiation through enhancing glycolysis without a significant effect on mitochondrial OCR.^7^ Metformin treatment also improved glucose metabolism and bone mass in type 2 diabetic mice in vivo.^7^ In addition, resveratrol, a natural antioxidant, was shown to increase the osteogenic differentiation of human periosteum-derived MSCs (PO-MSCs) through promoting mitochondrial biogenesis.^45^ Resveratrol dose-dependently elevated mitochondrial DNA contents, mitochondrial mass, and osteogenesis in PO-MSCs.^45^

Several mitophagy enhancers were also proposed to exhibit osteo-stimulating effects. Rapamycin, an inhibitor of the mammalian target of rapamycin protein kinase, was recently reported to activate PINK1/Parkin-mediated mitophagy to reduce advanced oxidation protein product-stimulated apoptosis of MC3T3-E1 osteoblasts and increase bone mass in mice.^46^ Ferutinin, a strong non-steroidal phytoestrogen, was also shown to boost osteoblast differentiation of DPSCs by triggering mitophagy trough upregulation of PINK1/Parkin.^47^ Additionally, vitamin K2 elevated mitophagy and rescued dexamethasone-induced downregulation of rat osteoblast function in vitro and bone mass in vivo, highlighting the therapeutic potential of vitamin K2 supplementation in alleviating glucocorticoid-induced osteoporosis.^48^ These findings suggest that compounds promoting mitophagy may have potential as therapeutic agents for enhancing osteoblast function and bone formation.

Despite the significant impact of mitochondrial morphology on cellular differentiation, evaluation of mitochondrial morphology regulators in osteoblasts is relatively scarce. Recently, our group reported that inducing mitochondrial fragmentation during the early osteoblast maturation process accelerated osteogenesis, while mitochondrial fusion promoter M1, which was suggested as a potential therapeutic agent for diabetic cardiomyopathy and neurodegenerative diseases, significantly impaired osteoblast activity and bone formation in mice.^2^ Our group also demonstrated that bongkrekic acid enhanced mitochondrial fragmentation and donut formation in mouse osteoblasts, thereby promoting their maturation.^2^ Triggering mitochondrial fragmentation and donut formation elevated extracellular mitochondrial secretion, which stimulated the differentiation of osteoprogenitors, and treatment with secreted mitochondria and MDVs significantly accelerated bone regeneration in mice.^2^ The potential of mitochondrial transplantation therapy for bone regeneration has recently been discussed by our group.^1^ Moreover, treatment of non-lipotoxic levels of 25 μM PA with glucose significantly increased osteogenic differentiation and calcification in MC3T3-E1 and primary osteoblasts by enhancing mitochondrial fission through the activation of mitochondrial fission protein DRP1, while treatment with mitochondrial division inhibitor mdivi-1 reduced osteogenesis.^26^ Collectively, the modulation of mitochondrial dynamics is a promising avenue for interventions aimed at regulating osteogenesis, but further examinations of targeting mitochondrial morphology including mitochondrial donut formation in osteoblasts under different conditions and possible side effects are necessary.

Various antioxidants were suggested to stimulate osteoblast activity in vitro and recently reviewed.^49^ Some of the major antioxidants described to rescue osteoblast function from oxidative stress include NAC, resveratrol, vitamin C, NAM, α-Lipoic acid (α-LA), and mitoquinone (MitoQ).^50^ However, variable results exist regarding the in vivo effects of antioxidant treatment on bone. For instance, MitoQ, a mitochondria-targeted antioxidant, was shown to prevent the loss of BMD and osteoblast function in diabetic and obese mice by reducing mitochondrial ROS generation in osteoblasts.^50^ On the other hand, Poudel et al. more recently demonstrated that MitoQ treatment via food in mice aged from 4 to 22 mo had no significant effect on aging-related reductions in vertebral and femoral bone mass, describing that targeting oxidative stress may not be an ideal strategy for countering aging-related decreases in bone health.^51^ However, only a single dose level of MitoQ was assessed in the study, generating the possibility that different doses may result in a positive outcome. The variable results observed in different studies highlight the complexity of antioxidant effects on bone health and imply that the outcomes may be influenced by factors such as dosage, treatment duration, and specific conditions such as aging and diabetes. Further research is needed to elucidate the optimal conditions for antioxidant therapy to positively impact bone health.

## Conclusions and future perspectives

In this comprehensive review, we explored various facets of mitochondria, including energy production, biogenesis, dynamics, and oxidative stress, and their impact on osteoblast function. Mitochondria in osteoblasts exhibit distinct metabolic, morphological, and secretory features that make them potential therapeutic targets. Key observations during osteoblast maturation involve the increase in glycolysis, mitochondrial fragmentation and donut formation, extracellular mitochondrial secretion, and mitophagy. Pharmacological agents that stimulate these activities generally promote osteogenic differentiation and bone formation, while inhibitors exhibit the opposite effects. Nevertheless, certain crucial aspects of mitochondria in osteoblasts need further investigation to facilitate the development of novel bone anabolic agents targeting osteoblast mitochondria. For example, further examination is needed to explore the mechanisms and functional significance of mitochondrial donut formation, its prevalence in osteoblasts of different origins, and the molecular pathways governing extracellular mitochondrial secretion. Also, mitochondrial characteristics in osteogenically-differentiating BMSCs should be established using a more homogeneous cell population, and whether mitochondrial patterns vary based on the embryonic origin of osteogenic cells needs clarification. Finally, and importantly, osteocytes and osteoclasts, other major bone cells, have gained increasing attention for their mitochondrial functions. Although not covered in this review, elucidating mitochondrial signatures in osteocytes and osteoclasts will contribute to optimizing mitochondrial therapeutics targeting both bone formation and resorption.

## Author contributions

Joonho Suh (Conceptualization; writing—original draft; writing—review and editing) and Yun-Sil Lee (Conceptualization; funding acquisition; supervision; writing—original draft; writing—review and editing)

## Funding

This work was supported by National Research Foundation of Korea (NRF) grants (NRF-2018R1A5A2024418, RS-2023-00246115, and RS-2024-00336924).

## Conflicts of interest

The authors declare that they have no conflicts of interest.

## Data availability

N/A.
